# Anti-PD-1 exacerbates bleomycin-induced lung injury in mice via Caspase-3/GSDME-mediated pyroptosis

**DOI:** 10.1038/s41419-024-07319-9

**Published:** 2025-01-06

**Authors:** Fei Wang, Haiyi Deng, Maolin Zhou, Yilin Yang, Jiankui Zhou, Yansheng Wang, Xiaohong Xie, Xinqing Lin, Ming Liu, Gengyun Sun, Chengzhi Zhou

**Affiliations:** 1 Department of Respiratory and Critical Care Medicine, the First Affiliated Hospital of Anhui Medical University, Anhui, 230022 China; 2 State Key Laboratory of Respiratory Diseases, National Clinical Research Center for Respiratory Disease, National Center for Respiratory Medicine, Department of Pulmonary and Critical Care Medicine, Guangzhou Institute of Respiratory Health, The First Affiliated Hospital of Guangzhou Medical University, Guangzhou, Guangdong, 510120 China; 3 Precise Genome Engineering Center, School of Life Sciences, Guangzhou University, Guangzhou, Guangdong 510405 China

**Keywords:** Cancer immunotherapy, Acute inflammation

## Abstract

Immune checkpoint inhibitors (ICIs) have significant therapeutic effects but can also cause fatal lung injury. However, the lack of mouse animal models of ICI-related lung injury (ICI-LI) has limited the in-depth exploration of its pathogenesis. In clinical practice, underlying lung diseases increase the risk of lung injury. Thus, we used a mouse model of lung injury induced by bleomycin (BLM) and then administered anti-programmed cell death 1 (aPD-1) antibodies to induce ICI-LI. Compared with the BLM group, the aPD-1 + BLM group presented more significant weight loss, greater levels of lung inflammation and fibrosis, and decreased lung function. In this ICI-LI model, high levels of caspase-3/gasdermin E (GSDME) were detected in the lung tissue of mice, and the JNK inhibitor SP600125 mitigated lung damage by inhibiting GSDME-mediated pyroptosis. Consistent with the findings in the animal model, immunofluorescence and RNA sequencing of lung tissue from ICI-LI patients revealed upregulation of the expression of genes related to the GSDME-related pyroptosis pathway. Our results suggest that GSDME-mediated pyroptosis may be associated with the pathogenesis of ICI-LI, indicating that targeting GSDME could be a potential therapeutic strategy for treating ICI-LI.

## Introduction

Recently, immune checkpoint inhibitors (ICIs) have been integrated into the standard management of solid cancers because of their remarkable potential in clinical trials. However, ICIs are associated with inflammatory side effects resulting from enhanced activity of the immune system, which are termed immune-related adverse events (irAEs) [1]. Pneumonitis is one of most common severe and fatal irAEs. According to a prospective study, the respiratory system is the most commonly affected site of severe irAEs and has the highest mortality rate (26.3%) [2]. However, the underlying mechanism is not yet clear. Current animal models of irAEs focus mainly on multiple organ dysfunction and require the use of gene-edited mice for modeling [3, 4]. However, animal models of pneumonitis are limited. Animal models that recapitulate pathophysiologic processes are urgently needed to identify therapeutic targets and develop strategies to mitigate the occurrence of severe ICI-LI. However, an anti-programmed cell death 1 (aPD-1) antibody alone in normal mice is not sufficient to induce pneumonitis [5]. Multiple studies have indicated that preexisting pulmonary fibrosis/interstitial lung disease is a risk factor for ICI-related lung injury (ICI-LI) [6, 7]. Therefore, the addition of an anti-PD-1 antibody in addition to bleomycin-induced lung injury may induce an ICI-LI model. Pyroptosis is an inflammatory programmed cell death pathway characterized by gasdermin (GSDM) protein (e.g., GSDMD and GSDME) family-mediated membrane perforation and the consecutive release of proinflammatory cytokines (e.g., IL-1β and IL-18) [8]. Recent studies have highlighted the significant role that pyroptosis plays in lung injury [9–11]. Accumulating evidence has shown that pyroptosis plays a crucial role in the development of radiation-induced pneumonitis [12]. However, the relationship between pyroptosis and ICI-LI remains unclear.

Previous studies have shown that increased C-Jun N-terminal kinase (JNK) activation is associated with irAEs [13]. JNK is a member of the mitogen-activated protein kinase (MAPK) subfamily and is involved in the occurrence and development of various inflammatory diseases [14]. One study showed that the JNK inhibitor SP600125 could prevent GSDME-mediated pyroptosis [15]. Therefore, this study aimed to determine whether pyroptosis participates in the progression of ICI-LI and whether inhibiting JNK could reduce GSDME-mediated pyroptosis induced by bleomycin (BLM) and aPD-1 antibodies.

## Materials and methods

### Patients

We searched for patients who were diagnosed with ICI-LI and underwent pathological biopsy at the First Affiliated Hospital of Guangzhou Medical University between 2020 and 2022. Paraffin-embedded tissue blocks from these patients were carefully preserved for further analysis. The control group consisted of lung cancer patients who underwent surgical treatment, and their postoperative paracancerous tissue paraffin samples were retained for analysis. The present study was approved by the Ethics Committee of the First Affiliated Hospital of Guangzhou Medical University. Written informed consent was obtained from all patients enrolled in this study.

### Mice

Six- to eight-week-old male C57BL/6 wild-type mice were obtained from Zhaoqing Ruisiyuan Biotechnology Co., Ltd. (Zhaoqing, China), housed at a constant temperature (22 ± 0.5 °C) under a 12/12 h light/dark cycle and fed autoclaved food and water at the Experimental Animal Centre of Guangzhou Medical University (Guangzhou, China).

### Mouse treatment procedures

The mice were allocated randomly into three groups: the vehicle control group, BLM group, and BLM+aPD-1 group (each group with 15 animals). The BLM group was subjected to a single intratracheal instillation of bleomycin (3 mg/mL; Nippon Kayaku, Japan) at 2 mg/kg (Supplementary Fig. S1A). In the BLM+aPD-1 group, the mice received both a single intratracheal instillation of bleomycin and an intraperitoneal injection of an anti-mouse PD-1 mAb (clone: RMP1-14, BioXcell) at 200 µg/dose (Supplementary Fig. S1B) every three days for a total of 15 days.

The mice were then divided into three groups: the vehicle-only control group, the BLM+aPD-1 group, and the BLM+aPD-1 + SP600125 group. In the BLM+aPD-1 + SP600125 group, the mice receiving BLM and anti-PD-1 treatment were intraperitoneally administered SP600125, which was dissolved in 2% dimethyl sulfoxide (DMSO) in phosphate-buffered saline (PBS), at a dosage of 30 mg/kg body weight once daily [15]. In the BLM+aPD-1 group, the mice received not only the aforementioned BLM and anti-PD-1 mAbs but also an equal volume of vehicle solution (DMSO or PBS).

### Histological, immunohistochemical, and immunofluorescence analyses

The lung tissue was carefully excised and weighed using an electronic balance. Lungs were fixed with 10% formalin and embedded in paraffin. Tissue sections (5 μm thick) were obtained for hematoxylin and eosin (H&E) staining, Masson staining and immunohistochemistry. After deparaffinization and rehydration of the paraffin sections, antigen retrieval was performed with sodium citrate buffer. For immunohistochemical (IHC) analysis, the sections were incubated with 3% hydrogen peroxide for 10 minutes and then blocked with 1% sheep serum. Mouse or human lung sections were subjected to overnight incubation at 4 °C with primary antibodies, including anti-α-smooth muscle actin (α-SMA) (catalog no. bs-10196R; Bioss), anti-GSDMA (catalog no. ab230768; Abcam), anti-GSDMB (catalog no. ab235540; Abcam), anti-GSDMC (catalog no. NBP1-91926; Novusbio), anti-GSDMD (catalog no. NBP2-33422; Novusbio), anti-GSDME (catalog no. ab215191; Abcam), anti-IL-1β (catalog no. ab254360; Abcam), anti-caspase 3 (catalog no. 341034; Zenbio), anti-EPCAM (catalog no. A19301; ABclonal) and anti-CD68 (catalog no. ab955; Abcam) antibodies. For staining with secondary antibodies, goat anti-rabbit IgG (1:10,000, Earthox, #720381) was used. 3,3’-Diaminobenzidine tetrahydrochloride (DAB) served as the chromogen for immunohistochemistry. For immunofluorescence staining, the cell nuclei were labeled with DAPI (catalog no. C1006; Beyotime), goat anti-rabbit Ig Ab (1:400, catalog no. 5220-0336; SeraCare) and goat anti-mouse Ig Ab (1:400, catalog no. 5220-0341; SeraCare) were used as the secondary Ab. The images of immunohistochemical staining were scanned with a 3DHISTECH Case Viewer (Pannoramic SCAN, Hungary). Histological assessments were conducted by two pathologists who were blinded to the experimental details. Histopathological changes were evaluated using an optical microscope, with fixed random fields of view selected from each section.

### Western blot analysis

Proteins were extracted from lung tissues using radioimmunoprecipitation assay (RIPA) buffer (Beyotime, China) supplemented with 1% phenylmethylsulfonyl fluoride (PMSF; Beyotime, China). Total protein was quantified using a bicinchoninic acid (BCA) protein quantification kit (Vazyme, Wuhan, China). Then, 20 μg from each sample was separated on an SDS‒PAGE gel and subsequently transferred to a PVDF membrane (Millipore, USA). The membranes were blocked for 1 h in 5% bovine serum albumin (BSA) at room temperature and incubated overnight at 4 °C with rabbit anti-mouse GSDME (catalog no. ab215191; Abcam) and GAPDH (1:1,000, ABclonal, Wuhan, China) antibodies. The blots were developed with horseradish peroxidase (HRP)-conjugated anti-mouse or anti-rabbit whole IgG secondary antibodies (1:5,000, Abcam Biotechnology, Cambridge, UK). The ECL-Plus detection kit was purchased from Vazyme (Wuhan, China). Protein band detection was performed by using a Tanon-5200 infrared scanning system (Anon Science & Technology Co., Ltd., Shanghai, China). The intensity was analyzed by ImageJ.

### Quantitative PCR analysis

Total RNA was isolated from mouse lung tissue using TRIzol Reagent (TransGen Biotech, Beijing, China) and then reverse transcribed to cDNA with HiScript III RT SuperMix for qPCR ( + gDNA wiper). Quantitative real-time PCR analysis was performed on a QuantStudio (TM) 6 Flex System (Thermo Fisher Scientific, UK). Detailed information on the primer pairs is included in Supplementary Table S1. Gene expression of the target genes was analyzed using the 2-ΔΔCT method, with GAPDH serving as the internal standard for normalization.

### RNA sequencing analysis

RNA sequencing (RNA-Seq) was performed on total RNA extracted from whole-lung tissues of each mouse group. Total RNA was extracted for RNA-Seq by using the VAHTS Universal V6 RNA-seq Library Prep Kit (NR604-01/02). The concentration and quality of the extracted total RNA were evaluated using a 2100 RNA Nano 6000 Assay Kit (Agilent Technologies, CA, USA). Sequencing was performed by a sequencing service company (Annoroad, Beijing, China) using the Illumina sequencing platform.

### Transcriptome sequencing analysis

A standardized pipeline utilizing STAR and RSEM software was used to analyze tissue transcriptomes [16]. The quantification of gene RNA expression values was conducted based on the GRCm39 version of the gene annotation (https://www.gencodegenes.org/mouse/). Differential gene expression analysis was performed using the DESeq2 package [17]. Gene set enrichment analysis (GSEA) for Gene Ontology (GO) biological process (BP) terms was performed with the fgsea package. Volcano plots illustrating differentially expressed genes were generated using the ggplot2 package. The presentation of gene expression via heatmaps was achieved with the heatmap package. All analyses were performed on a Linux platform using R version 4.3.1 for optimal computational performance.

### Pulmonary function test

Pulmonary function was determined using the forced pulmonary maneuvering system (Buxco Research Systems, USA) according to the manufacturer’s protocol. Briefly, the mice were anesthetized with 1.2% Avertin solution and transferred to the body chamber of the system (Supplementary Fig. S1C). Lung function parameters such as dynamic compliance and respiratory resistance were measured.

### Chest computed tomography (CT)

Seven days after treatment, lung computed tomography (CT) was performed using a small-animal CT system, Super Nova Micro CT Type: SNC-100 (PINGSENG Healthcare, Kunshan, China). The mice were sedated, fixed in a supine position in a CT machine and imaged with the following parameters: imaging condition, lung; pixel size, 35 μm; slice thickness, 50 μm; slice interval, 50 μm; X-ray voltage, 230 V ~ ; and respiratory synchronization, “Yes”. The CT images were analyzed with a RadiAnt DICOM Viewer (Medixant, Poznan, Poland).

### Statistical analysis

All the numerical data are presented as the means ± standard deviations. Differences among various groups were compared using one-way ANOVA with Tukey’s post hoc test, two-tailed unpaired Student’s t test or the Mann‒Whitney U test. The lung coefficient was calculated using the following formula: wet lung weight (g) divided by total body weight (kg). GraphPad Prism 8.0 and SPSS 25.0 software were used for graphics and statistical analyses. The results were considered statistically significant at *p* < 0.05.

## Results

### Effects of simultaneous BLM and anti-PD-1 therapy on lung inflammation

**Fig. 1:**
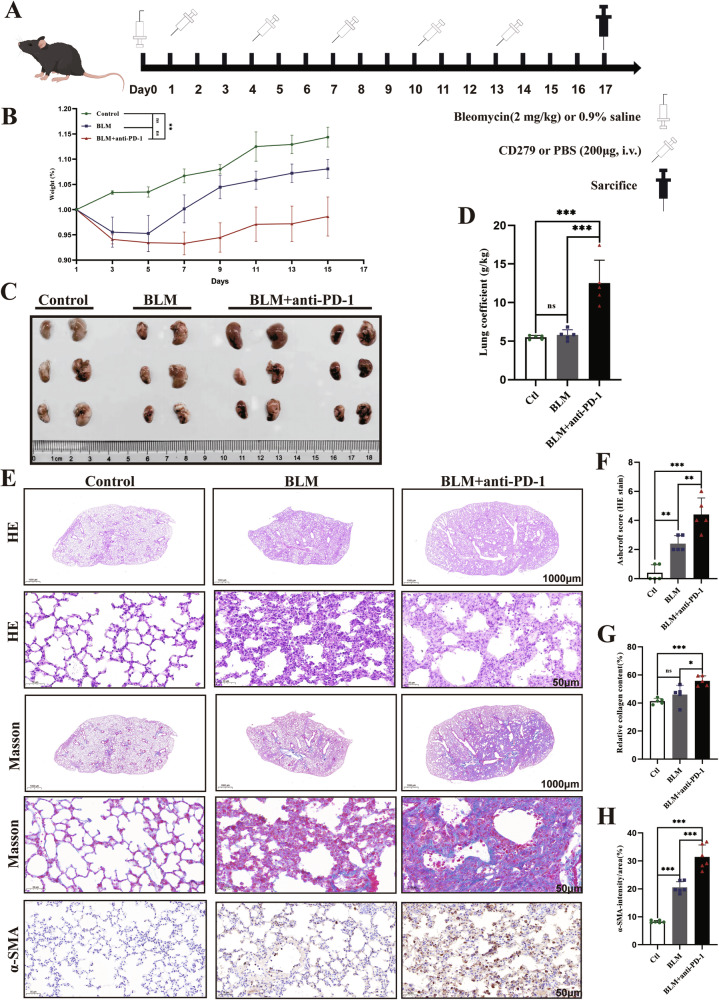
Effects of simultaneous BLM and anti-PD-1 therapy on lung inflammation. **A** Experimental scheme detailing the administration of BLM or BLM and the anti-PD-1 antibody. **B** Ratio of body weight changes to baseline in mice (*n* = 10/group). **C** Representative photomicrographs of lung tissue histopathology (*n* = 3–6/group). **D** Lung coefficient (*n* = 5/group). **E**–**H** Representative H&E (1000 μm, 50 μm) and Masson (1000 μm, 50 μm) staining of the indicated mouse lung sections and immunohistochemical staining for α-SMA in the mouse lung. The data are shown as the means ± SDs; ns, not significant, **p < 0.01, ***p < 0.001. α-SMA α-smooth muscle actin, BLM bleomycin, Ctl control, PD-1 programmed cell death 1.

The mice were treated with BLM alone or in combination with an anti-PD-1 antibody, as shown in Fig. 1A. We observed transient weight loss followed by recovery in the BLM group, whereas the BLM+anti-PD-1 group consistently had lower body weights than did the baseline group and significantly lower body weights than did the control group (Fig. 1B). On Day 17, the mice were euthanized, and their lungs were collected for histological analysis to assess the extent of inflammation. The BLM+aPD-1 group exhibited more pronounced lung edema, hyperemia, and congestion (Fig. 1C). The combined group exhibited a more pronounced increase in the lung coefficient (Fig. 1D). The lungs of the mice in the BLM group showed inflammatory cell infiltration and patchy fibrotic lesions, whereas those in the combination group showed more severe pulmonary inflammation and pathological damage with symptoms of interstitial lymphocyte infiltration in the lungs (Fig. 1E and F). Consistently, Masson’s trichrome staining revealed obvious collagen deposition in the lung interstitial area in the BLM+aPD-1 group (Fig. 1E and G). α-SMA is a useful clinical sign of pulmonary fibrosis. Compared with those in the control group, both the combined treatment group and the BLM group exhibited elevated levels of α-SMA in the lung tissue, with the combined group showing a more pronounced increase (Fig. 1E and H). These data indicate that PD-1 inhibitors exacerbate bleomycin-induced lung injury.

### CT imaging and lung function

**Fig. 2:**
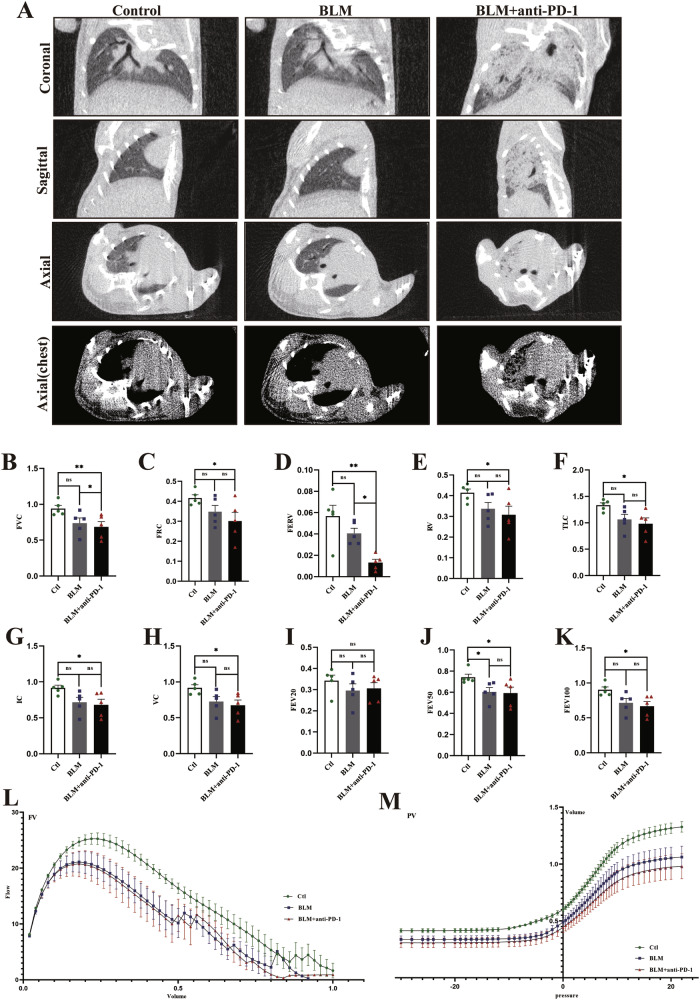
Computed tomography (CT) lung images and lung function in each group. **A** Representative coronal images and their corresponding sagittal and axial images from different groups were obtained using micro-CT (n = 4/group). **B**–**K** Forced volume capacity (FVC), functional residual capacity (FRC), forced expiratory reserve volume (FERV), residual volume (RV), total lung capacity (TLC), inspiratory capacity (IC), volume capacity (VC), forced expiratory volume at 20 ms (FEV20), forced expiratory volume at 50 ms (FEV50), and forced expiratory volume at 100 ms (FEV100) in all groups (*n* = 5/group). **L**–**M** Representative median flow‒volume (F‒V) and pressure‒volume (P‒V) curves for each group (*n* = 5/group). The data are shown as the means ± SDs; ns, not significant, * *P* < 0.05, **p < 0.01, ***p < 0.001.

On Day 7, the mice underwent successive chest CT. Compared with those in the control group, lung images in the BLM group revealed some infiltrative shadows (Fig. 2A). Compared with those of the other two groups, the lung images of the BLM+anti-PD-1 group showed increased density, along with diffuse ground‒glass opacities with or without areas of consolidation (Fig. 2A). These CT imaging changes were consistent across the different groups of mice, although quantitative evaluation was challenging.

The parameters included in the lung function test analysis are presented in Fig. 2. Compared with those in the other groups, the forced volume capacity (FVC), functional residual capacity (FRC), forced expiratory reserve volume (FERV), residual volume (RV), total lung capacity (TLC), inspiratory capacity (IC), volume capacity (VC), forced expiratory volume at 20 ms (FEV20), forced expiratory volume at 50 ms (FEV50), and forced expiratory volume at 100 ms (FEV100) in the BLM+aPD-1 group tended to decrease (Fig. 2B-K). The pulmonary flow‒volume (F‒V) and pressure‒volume (P‒V) curves demonstrated that both the BLM group and the BLM+aPD-1 group exhibited lower values than did the control group (Fig. 2L and M). These results suggest that the combined group had a more significant decline in lung function.

### BLM combined with a PD-1 agonist enhances pyroptosis

**Fig. 3:**
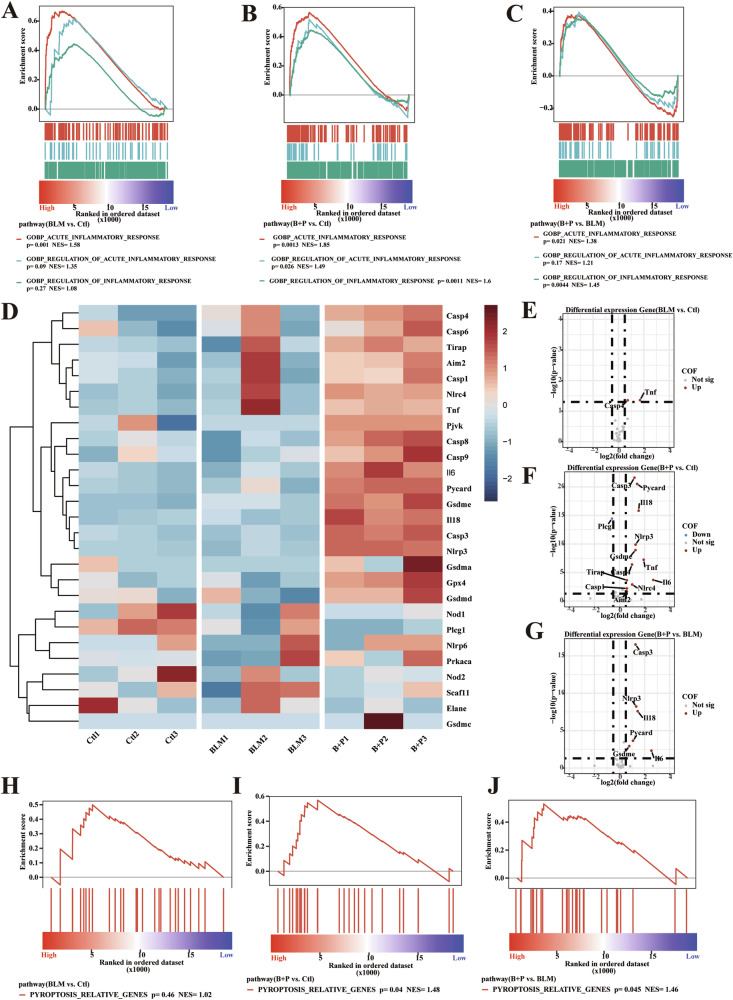
Molecular pathways associated with pneumonia injury in experimental mice. **A**–**C** GSEA plot of lung tissue among the groups. Gene ranking is based on the fold change in differential expression of acute inflammatory response-related pathways, arranged from high to low rank. **D** Heatmap of the expression of pyroptosis-related genes and the genes with the highest expression among the groups. Gene expression levels are scaled from -3 to 3. **E**–**G** Volcano plot showing the differential expression of pyroptosis-related genes among the groups. GSEA plot of lung tissue among the groups. **H**–**J** Gene ranking is based on the fold change in differential expression of pyroptosis pathways, arranged from high to low rank. GSEA, gene set enrichment analysis. NES, normalized enrichment score.

To determine whether pyroptosis occurs in BLM- and anti-PD-1-induced lung fibrosis, we first analyzed the RNA-seq results of mouse lungs. Both the BLM and BLM+aPD-1 treatment groups exhibited aggravated acute inflammatory responses in vivo (Fig. 3A-B). Notably, the BLM+aPD-1 group exhibited a more pronounced inflammatory response than the BLM monotherapy group did, indicating that BLM had enhanced regulatory effects on inflammation (Fig. 3C). Furthermore, the BLM+aPD-1 group exhibited increased expression of pyroptosis-related genes (Fig. 3D; Supplementary Fig. S2). Differential expression analysis revealed that, compared with that in the control group, there was no significant difference in GSDME-related apoptosis in the bleomycin group (Fig. 3E). In contrast, the expression of GSDME, caspase 3, IL-6, and IL-18 was greater in the BLM+aPD-1 group than in the control group (Fig. 3F) and the BLM monotherapy group (Fig. 3G). Moreover, pyroptosis-related pathways were significantly enriched in the BLM+aPD-1 group (*p* = 0.04), in contrast to the nonsignificant enrichment in the BLM monotherapy group (*p* = 0.46) (Fig. 3H-J). These findings suggest that combined therapy with BLM and aPD-1 may induce pyroptosis, thereby intensifying the inflammatory response.

### Upregulation and activation of GSDME in mice treated with BLM and anti-PD-1 antibodies

**Fig. 4:**
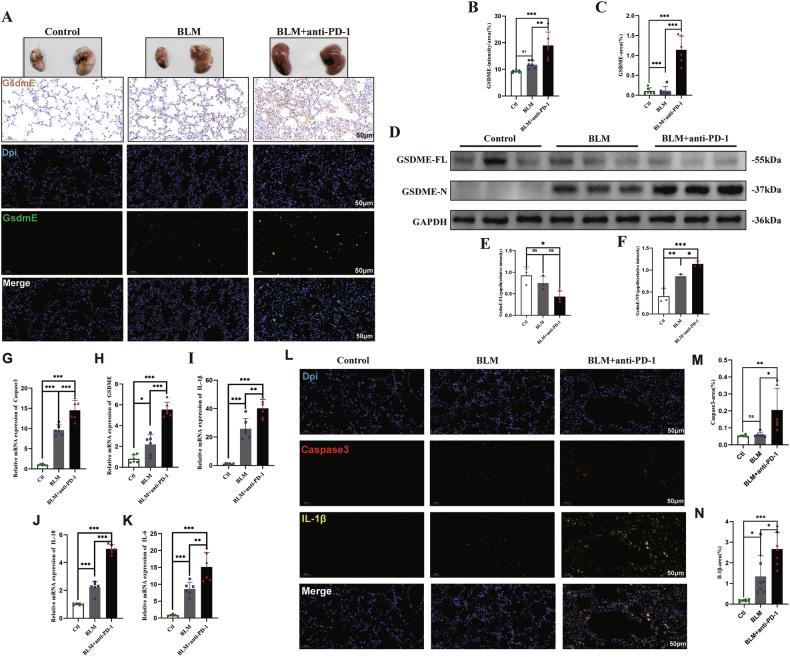
Upregulation and activation of GSDME in mice treated with BLM and an anti-PD-1 antibody. **A**–**C** Immunohistochemical and immunofluorescence analyses of the expression of GSDME in lung samples; **D**–**F** Western blot and quantitative analysis of full-length GSDME and cleaved GSDME in the indicated groups (*n* = 3/group); **G**–**L** Relative mRNA expression levels of GSDME, caspase-3, IL-1β, IL-6, and IL-18 (*n* = 6/group); **M**–**O** Immunofluorescence analysis of the expression of caspase-3 and IL-1β in lung samples. The data are shown as the means ± SDs; ns, not significant, * *P* < 0.05, **p < 0.01, ***p < 0.001. BLM bleomycin, Ctl control, GSDME gasdermin E, PD-1 programmed cell death 1.

We used immunohistochemical staining to test for the presence of cleaved GSDME and found that GSDME was significantly upregulated in the BLM+aPD-1 group (Fig. 4A-B). Moreover, immunofluorescence staining revealed similar results: BLM+aPD-1 induced stronger green-stained GSDME signals (Fig. 4A and C). Western blot analysis revealed that the level of full-length GSDME was reduced and that the level of GSDME-N was elevated in the combined therapy group (Fig. 4D-F). The lung samples were then used for RNA and protein extraction. qPCR revealed that after BLM and anti-PD-1 treatment, the transcription levels of caspase-3, GSDME, IL-1β, IL-18, and IL-6 increased (Fig. 4G-K). Double immunofluorescence staining revealed that red-stained cleaved caspase-3-positive signals and yellow-stained IL-1β signals were more intense in the BLM+aPD-1 group (Fig. 4L-N). These data indicate that GSDME-mediated pyroptosis is elevated and activated in mice treated with BLM and anti-PD-1 antibodies.

### SP600125 effectively alleviates pulmonary fibrosis in mice

**Fig. 5:**
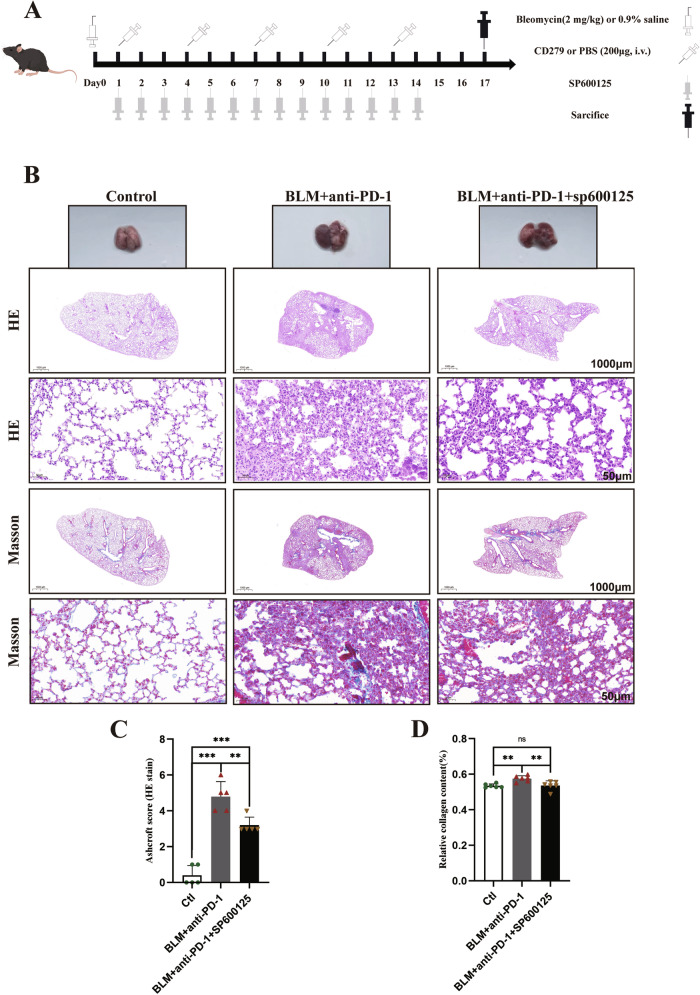
SP600125 effectively alleviates pulmonary fibrosis in mice. **A** Experimental scheme detailing the administration of BLM, PD-1 antibody and SP600125. **B-D** Representative H&E staining and Masson staining of the indicated mouse lung sections. The data are shown as the means ± SDs; ns, not significant, * *P* < 0.05, **p < 0.01, ***p < 0.001. BLM bleomycin; Ctl control; GSDME, gasdermin E; PD-1, programmed cell death 1.

To determine whether SP600125 could alleviate pulmonary fibrosis, we treated the mice with SP600125 for 2 weeks (Fig. 5A). HE staining of the lungs demonstrated that SP600125 treatment attenuated pulmonary inflammation and injury (Fig. 5B-C). Similarly, Masson’s trichrome staining revealed that SP600125 treatment reduced collagen deposition (Fig. 5B and D). Taken together, these findings indicate that SP600125 treatment can alleviate pulmonary fibrosis induced by BLM and aPD-1 in mice.

### SP600125 inhibits GSDME-mediated pyroptosis in mice

**Fig. 6:**
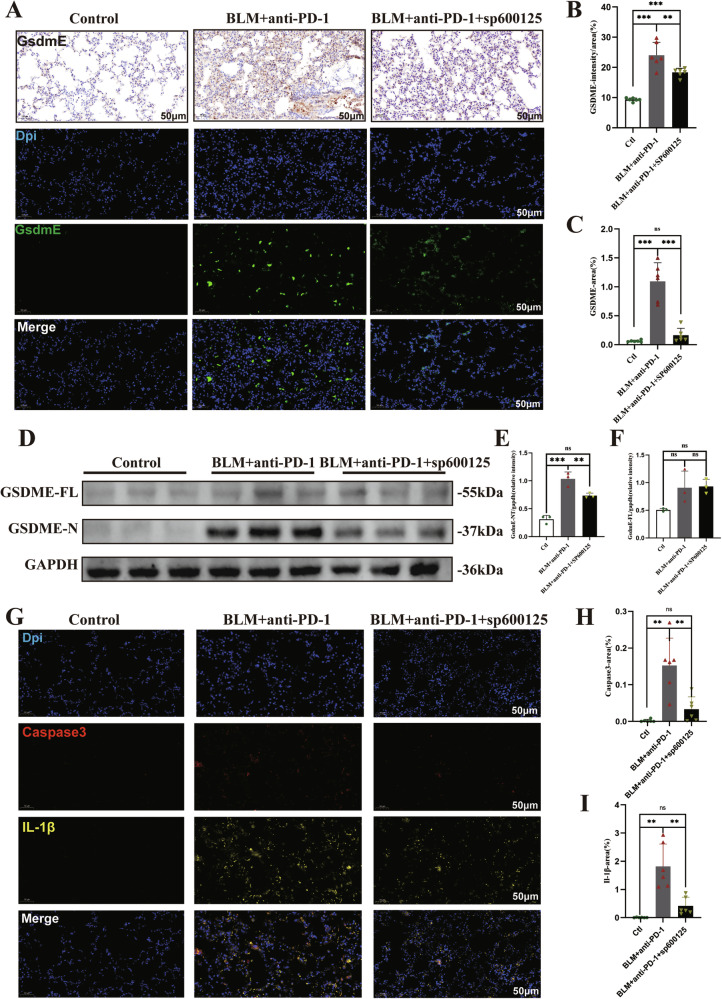
SP600125 inhibits GSDME-mediated pyroptosis in mice. **A**–**C** Immunohistochemistry and immunofluorescence staining of lung tissue with an anti-cleaved GSDME antibody; **D**–**F** Western blot and quantitative analysis of full-length GSDME and cleaved GSDME in the indicated groups. **G**–**I** Immunofluorescence analysis of the expression of caspase-3 and IL-1β in lung specimens. The data are shown as the means ± SDs; ns, not significant, * *P* < 0.05, **p < 0.01, ***p < 0.001. BLM bleomycin; Ctl control; GSDME gasdermin E; PD-1 programmed cell death 1.

A previous study revealed that the JNK inhibitor SP600125 effectively impeded GSDME-mediated pyroptosis [15]. Compared with that in the control group, the expression of GSDME in the lung was significantly elevated in the BLM+aPD-1 group (Fig. 6A-C). Notably, the mice with pulmonary fibrosis that were treated with SP600125 exhibited reduced protein expression levels of GSDME (Fig. 6A-C). Specifically, abundant levels of GSDME-N in the lung were easily detected in the BLM+aPD-1 group, whereas GSDME-N was significantly reduced in the mice treated with SP600125 (Fig. 6D-F). Similarly, lung injury model mice treated with SP600125 exhibited reduced protein expression levels of cleaved caspase-3 and IL-1β (Fig. 6G-I). These findings indicate that SP600125 effectively inhibited GSDME-mediated pyroptosis in the lung.

### Increased GSDME expression in patients with ICI-LI

**Fig. 7:**
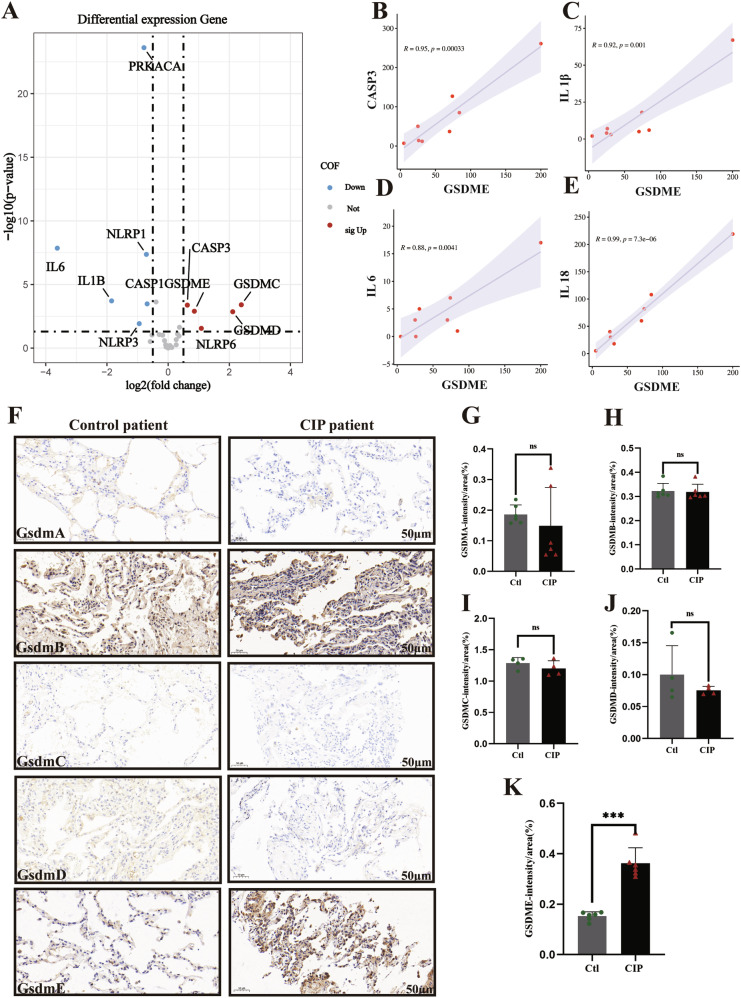
Increased GSDME expression in patients with ICI-LI. **A** A volcano plot depicting the differences in the expression of pyroptosis pathway components in each group. The red points represent the significantly upregulated genes. The blue points represent the significantly downregulated genes. The cutoffs used for the *p* value and |log2FC| for the differentially expressed genes were 0.05 and 1, respectively. **B**–**E** The correlation between GSDME mRNA expression levels and the mRNA expression levels of caspase 3, IL-1β, IL-18, and IL-6 in ICI-LI patients. **F**–**K** Representative images of lung tissue stained with antigasdermin antibodies.

To investigate the role of the pyroptosis executor GSDME in patients with ICI-LI, we first analyzed the RNA-seq data of lung tissue from our previous study [18], which included non-small cell lung cancer (NSCLC) patients treated with ICIs who either developed ICI-LI (*n* = 8) or did not develop ICI-LI (*n* = 29). The expression levels of GSDME and caspase-3 were significantly greater in ICI-LI patients than in non-ICI-LI patients (Fig. 7A). Additionally, GSDME was positively correlated with caspase 3, IL-1β, IL-6, and IL-18 (Fig. 7B-E) in the ICI-LI group. To determine whether GSDME-induced pyroptosis occurs in ICI-LI, we used immunohistochemical staining to determine whether cleaved GSDME was present in the lung tissue of ICI-LI patients. We enrolled a total of 10 patients whose paraffin-embedded lung tissues were used for research, including 5 patients with ICI-LI and 5 controls. The demographics and characteristics of these patients are shown in Supplementary Table S2 and Supplementary Fig. S3. GSDME expression in the lungs of patients with ICI-LI was significantly greater than that in the lungs of controls, but there were no significant differences in the expression of other gasdermin proteins (Fig. 7F-K). These data suggest that GSDME-mediated pyroptosis may play an important role in the pathogenesis and development of ICI-LI.

## Discussion

ICIs enhance treatment efficacy in cancer patients, but they can also lead to lung injury. Furthermore, the presence of pulmonary fibrosis increases the risk of lung injury in patients receiving immunotherapy. ICI-LI carries a risk of fatality, necessitating the establishment of appropriate animal models to further investigate the pathogenesis of ICI-LI. In this study, we did not observe obvious pneumonitis in normal C57BL/6 mice treated with PD-1 inhibitors only (Supplementary Fig. S4). However, the combined use of BLM and PD-1 antibodies is associated with increased inflammation in the lung tissue of mice. In addition to histopathology, we also assessed lung inflammation using chest CT scans and lung function tests. This is more in line with clinical practice, as biopsy pathology is not necessary for the diagnosis of ICI-LI.

Importantly, in the present study, we identified GSDME-mediated pyroptosis as a key driver of ICI-LI. Pyroptosis is a proinflammatory form of cell death that relies on the caspase family. In recent years, the caspase-3/GSDME-dependent pyroptosis signaling pathway has been identified. Previous studies have shown that GSDME activation can induce antitumor immunity and improve ICI efficacy [19] but may also lead to damage to normal tissues. Pyroptosis leads not only to abnormal cell death but also to the recruitment of immune cells to trigger an inflammatory cascade, leading to normal inflammatory cell death. One previous study demonstrated that CAR-T-cell therapy activates caspase-3/GSDME-dependent pyroptosis, resulting in cytokine release syndrome (CRS) [20]. Cytokine release and CRS also occur in patients with ICI-LI [21]. One recent study revealed that GSDME-related pyroptosis is associated with ICI-induced myocarditis [22]. Therefore, pyroptosis may be involved in the occurrence and development of ICI-LI. In this study, we first demonstrated that cleaved GSDME levels were increased in the lungs of mice with aPD-1 + BLM-induced lung fibrosis. In addition, there was increased expression of caspase-3 and increased release of IL-1β, IL-6, and IL-18 in the lungs of ICI-LI mice. Moreover, the expression of GSDME in the pneumonia lesions of ICI-LI patients was also increased, suggesting that GSDME-mediated pyroptosis may play an important role in the pathogenesis and development of ICI-LI. We did not find elevated IL-1β, or IL-18 in RNA-seq of lung tissues from ICI-LI patients. A possible explanation is that the control group was NSCLC patients treated with ICI rather than normal subjects. IL-1β and IL-18 are highly associated with cancer progression [23], and IL-1β might be related to immunotherapy resistance [24]. Multiple factors influence these cytokines, thus they may not serve as independent markers for evaluating pyroptosis. We performed immunofluorescence on the lung tissues of mice treated with combination therapy and ICI-LI patients and found that cleaved GSDME was upregulated in macrophages and nearby alveolar epithelial cells (Supplementary Fig. S5). We hypothesize that macrophages undergo pyroptosis first, promoting pyroptosis in nearby alveolar epithelial cells. Nevertheless, this assumption needs further investigation.

**Fig. 8:**
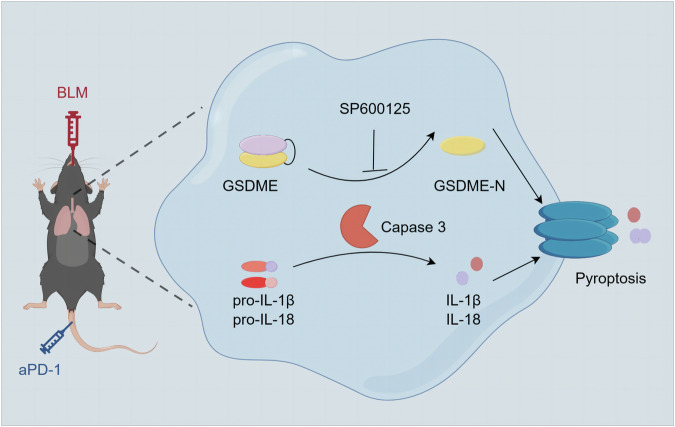
Schematic diagram of the mechanisms underlying BLM+anti-PD-1-induced pyroptosis. Anti-PD-1 exacerbates bleomycin-induced lung injury via caspase-3/GSDME-mediated pyroptosis, and SP600125 attenuates GSDME-mediated pyroptosis to alleviate lung injury.

Moreover, treatment with the JNK inhibitor SP600125 significantly decreased the expression of GSDME, caspase-3 and IL-1β in the lungs of ICI-LI mice. Importantly, SP600125 administration effectively ameliorated lung fibrosis in the ICI-LI mice. These results suggest that SP600125 effectively attenuates lung fibrosis in ICI-LI mice by inhibiting GSDME-mediated pyroptosis. Previous research indicates that dysregulation of the JNK pathway is associated with numerous inflammatory diseases and autoimmune conditions [14, 25]. Significantly increased JNK expression has been observed in patients with pulmonary fibrosis [26]. One study demonstrated that the JNK inhibitor SP600125 can effectively block GSDME-mediated pyroptosis [15]. Similarly, JNK inhibitors alleviate cisplatin- or doxorubicin-induced pyroptosis by inhibiting the activation of caspase-3 and GSDME [27]. Therefore, JNK inhibitors may have potential as therapeutic agents for treating ICI-LI by inhibiting GSDME-mediated pyroptosis. In summary, we found that the caspase3/GSDME pathway is essential for the development of ICI-LI, and SP600125 attenuates GSDME-mediated pyroptosis to alleviate ICI-LI (Fig. 8).

This study is the first to explore the role of GSDME in the pathogenesis of ICI-LI. However, the findings of the present study have limitations. The mechanism of ICI-LI is relatively complex and cannot be fully simulated via in vitro experiments. This problem may be solved by the development of in vitro technology and in-depth research on ICI-LI. In addition, studying the pyroptosis of specific cells will be helpful in understanding the pathophysiology of ICI-LI and requires further research.

## Conclusions

In conclusion, our study provides evidence that the combination of PD-1 inhibitors and BLM more readily induces lung fibrosis. We also revealed the significant involvement of GSDME-mediated pyroptosis in the pathogenesis and development of ICI-LI and demonstrated that the JNK inhibitor SP600125 can alleviate lung fibrosis by inhibiting GSDME-mediated pyroptosis.

## Supplementary information

Supplementary materials

Full and uncropped western blots

## Data Availability

All data needed to evaluate the conclusions in the paper are present in the paper and/or the Supplementary Materials. RNA-seq data is deposited in National Center for Biotechnology Information (NCBI Gene Expression Omnibus GSE280068). Additional data related to this paper may be requested from the corresponding author upon reasonable request.
