# Supplementary material for: Anti-PD-1 exacerbates bleomycin-induced lung injury in mice via Caspase-3/GSDME-mediated pyroptosis
Source: Cell Death Dis. 2025 Jan 6;16(1):3. doi: 10.1038/s41419-024-07319-9 (PMC11704276; doi:10.1038/s41419-024-07319-9)
Supplement: Supplementary file 1 — Supplementary materials [file 41419_2024_7319_MOESM1_ESM.docx]

**Supplementary Information**

Table S1 Sequences of primers used for targeted gene expression detection.

| Gene | Forward primer 5’-3’ | Reverse primer 5’-3’ |
| --- | --- | --- |
| mGSDME | TGCAACTTCTAAGTCTGGTGACC | CTCCACAACCACTGGACTGAG |
| mCaspase3 | GGCTTGCCAGAAGATACCGGT | GCATAAATTCTAGCTTGTGCGCGTA |
| mIL‐18 | ACTTCACTGTACAACCGCA | TCAGTCATATCCTCGAACAC |
| mIL-1β | GAAATGCCACCTTTTGACAGTG | TGGATGCTCTCATCAGGACAG |
| mIL-6 | CATGTTCTCTGGGAAATCGTGG | GTACTCCAGGTAGCTATGGTAC |

Table S2. Clinical information about the patients with ICI-LI and the controls.

| Group | ICI-LI group | | | | | Non-ICI-LI group | | | | |
| --- | --- | --- | --- | --- | --- | --- | --- | --- | --- | --- |
|  | P1 | P2 | P3 | P4 | P5 | N1 | N2 | N3 | N4 | N5 |
| Age | 49 | 78 | 65 | 58 | 63 | 72 | 55 | 59 | 64 | 45 |
| Sex | Male | Male | Male | Male | Male | Female | Male | Female | Female | Female |
| Smoking status | Yes | Yes | No | No | Yes | No | Yes | No | No | No |
| Histologic classification | Squ | Ade | Squ | Ade | Ade | Ade | Squ | Squ | Ade | Ade |
| WBCs(×10^9/L) | 4.40 | 9.2 | 11.6 | 8.2 | 8.3 | 6.0 | 9.2 | 11.7 | 10.2 | 10.7 |
| NEU (×10^9/L) | 3.7 | 6.1 | 9.4 | 5.4 | 6.4 | 3.9 | 7.0 | 11.1 | 9.4 | 9.8 |
| LYM (×10^9/L) | 0.4 | 2.2 | 0.5 | 1.5 | 1.1 | 1.2 | 1.4 | 0.5 | 0.5 | 0.4 |
| PLTs (×10^12/L) | 205 | 278 | 529 | 269 | 260 | 313 | 196 | 207 | 223 | 210 |
| IL-2 (pg/ml) | 1.29 | 0.19 | 1.2 | 1.97 | 0.72 | - | - | - | - | - |
| IL-4 (pg/ml) | 1.97 | 0.03 | 1.8 | 0.67 | 0.12 | - | - | - | - | - |
| IL-6 (pg/ml) | 309.7 | 14.67 | 2.48 | 8.02 | 1.01 | - | - | - | - | - |
| IL-10 (pg/ml) | 20.35 | 2.28 | 2.69 | 14.68 | 1.37 | - | - | - | - | - |
| IFN-γ (pg/ml) | 39.76 | 2.12 | 5.66 | 1.47 | 1.13 | - | - | - | - | - |
| TNF-α (pg/ml) | 0.63 | 1.10 | 3.42 | 1.25 | 0.93 | - | - | - | - | - |
| KL-6 (U/L) | 2638 | 1379 | - | 4112 | 3272 | - | - | - | - | - |
| CRP (mg/dL) | 83.74 | 18.12 | - | 2.21 | 13.67 | - | - | - | - | - |
| LDH (U/L) | 318 | 208.3 | 227.6 | 426 | 255.9 | - | - | - | - | - |
| ALB (g/L) | 34.3 | 36.7 | 24.3 | 36.8 | 42.9 | 38.8 | 43.9 | 37.5 | 44.3 | 32.3 |

Ade, adenocarcinoma; ALB, albumin; CRP, C-reactive protein; ICI-LI, immune checkpoint inhibitor-related lung injury; IFN-γ, [interferon](http://www.dictall.com/indu/317/31681743770.htm) γ; IL-2, interleukin-2; IL-4, interleukin-4; IL-6, interleukin-6; IL-10, interleukin-10; KL-6, Krebs Von den Lungen-6; LDH, lactate dehydrogenase; LYM, lymphocyte; PLTs, platelets; Squ, squamous; TNF-α, tumor necrosis factor-α; WBCs, white blood cells.


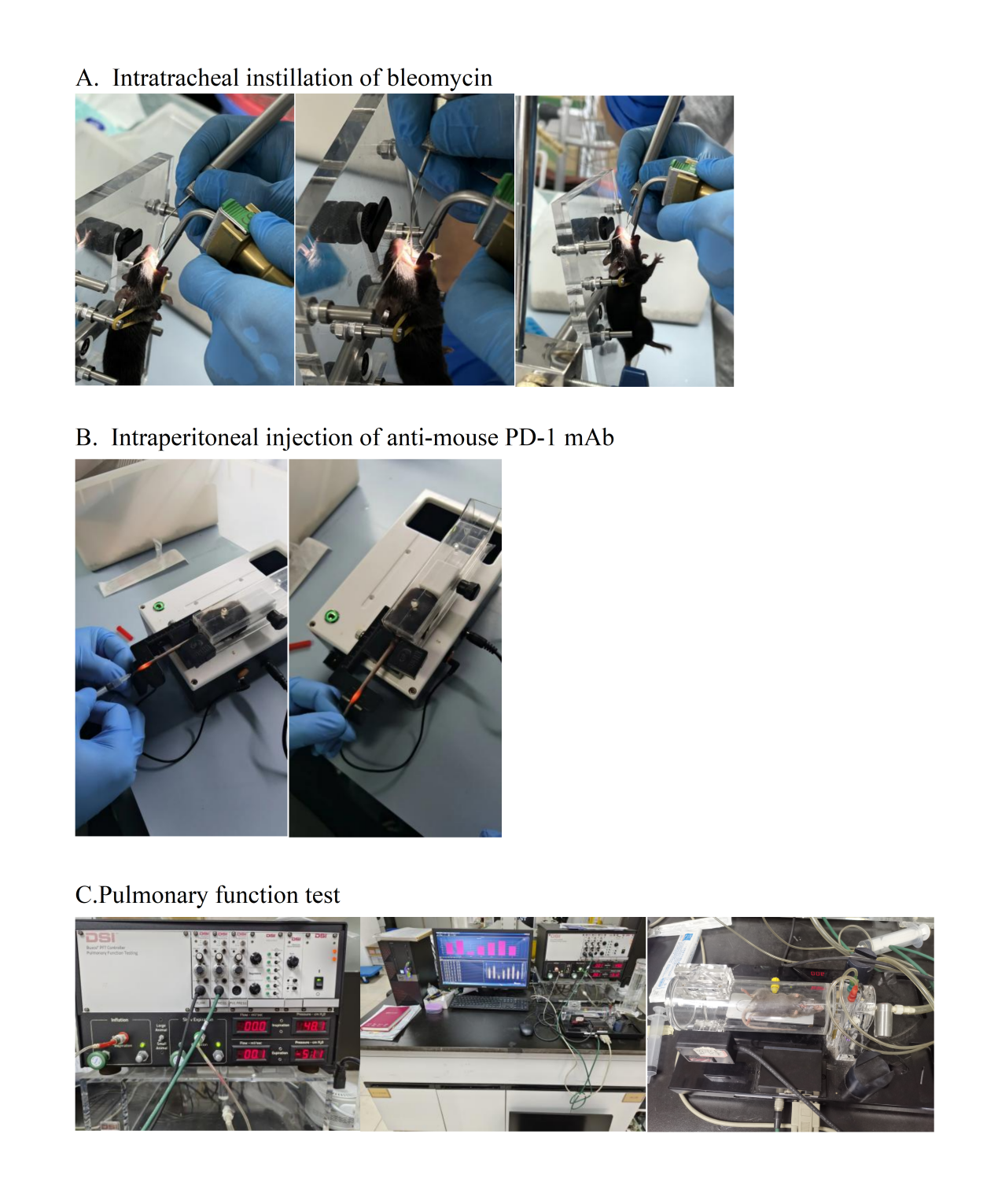


Supplementary Figure S1 Relevant operational images. (A) Intratracheal instillation of bleomycin; (B) Intraperitoneal injection of anti-mouse PD-1 mAb; (C) Pulmonary function test.


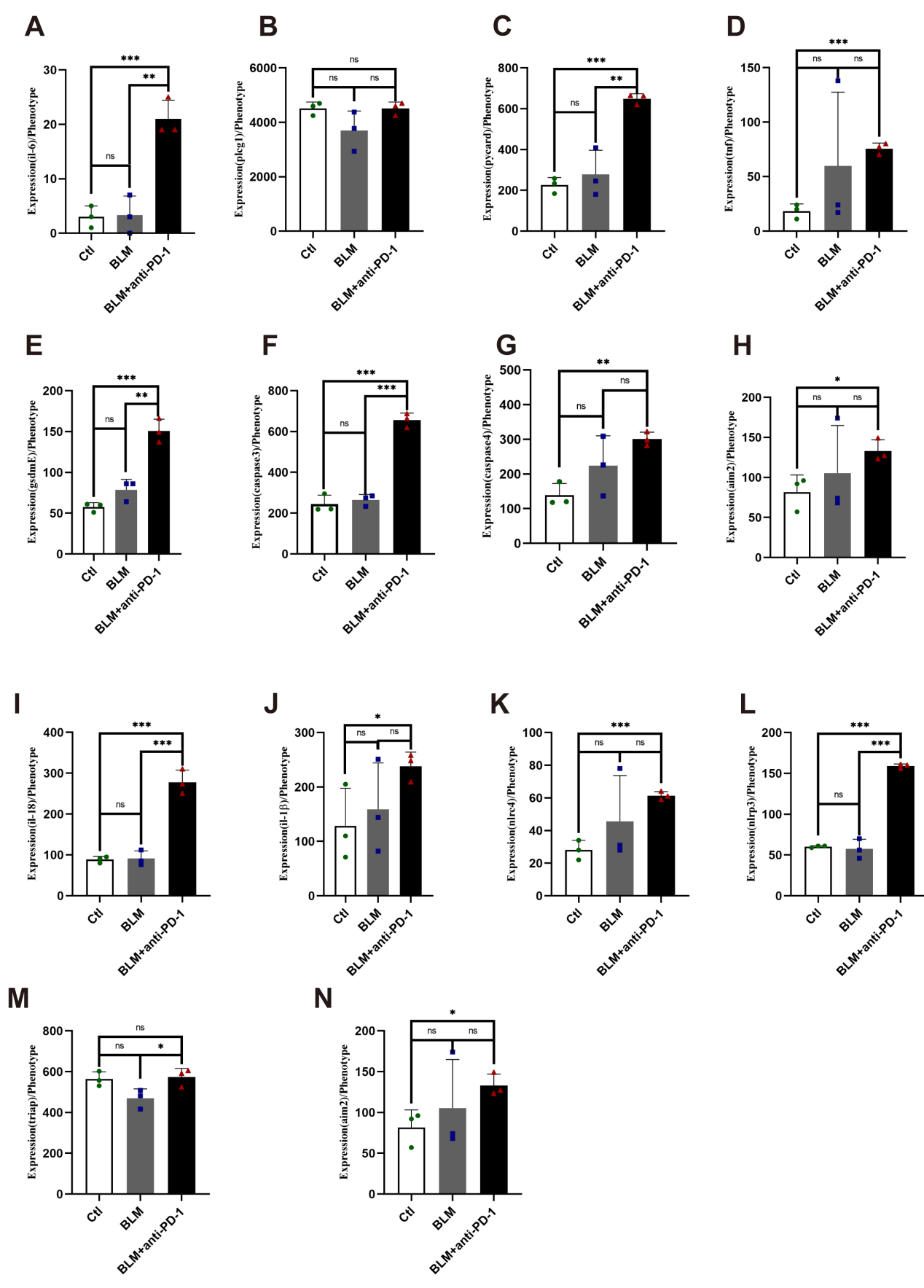


Supplementary Figure S2 Bar charts comparing the expression levels of pyroptosis-related genes among the groups. (A) IL-6; (B) Plcg; (C) Pycard; (D) Tnf; (E) Gsdme; (F) Caspase 3; (G) Caspase 4; (H) Aim2; (I) IL-18; (J) IL-1β; (K) Nlrc4; (L) Nlrp3; (M) Triap. The data are shown as the means ± SDs; ns, not significant, * P<0.05, **p < 0.01, ***p < 0.001.


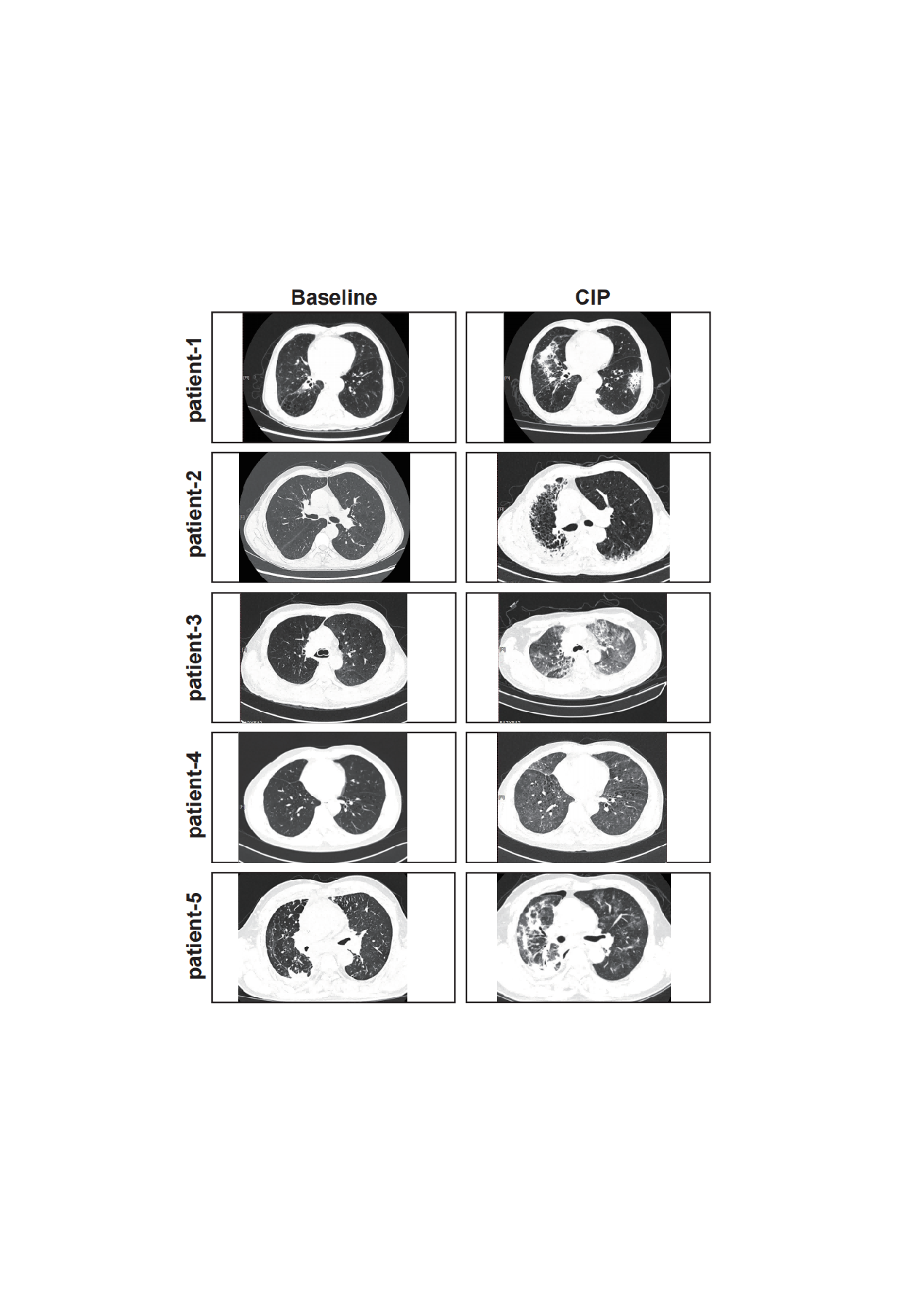


Supplementary Figure S3. Computed tomography (CT) lung images of patients with ICI-LI (n=5).


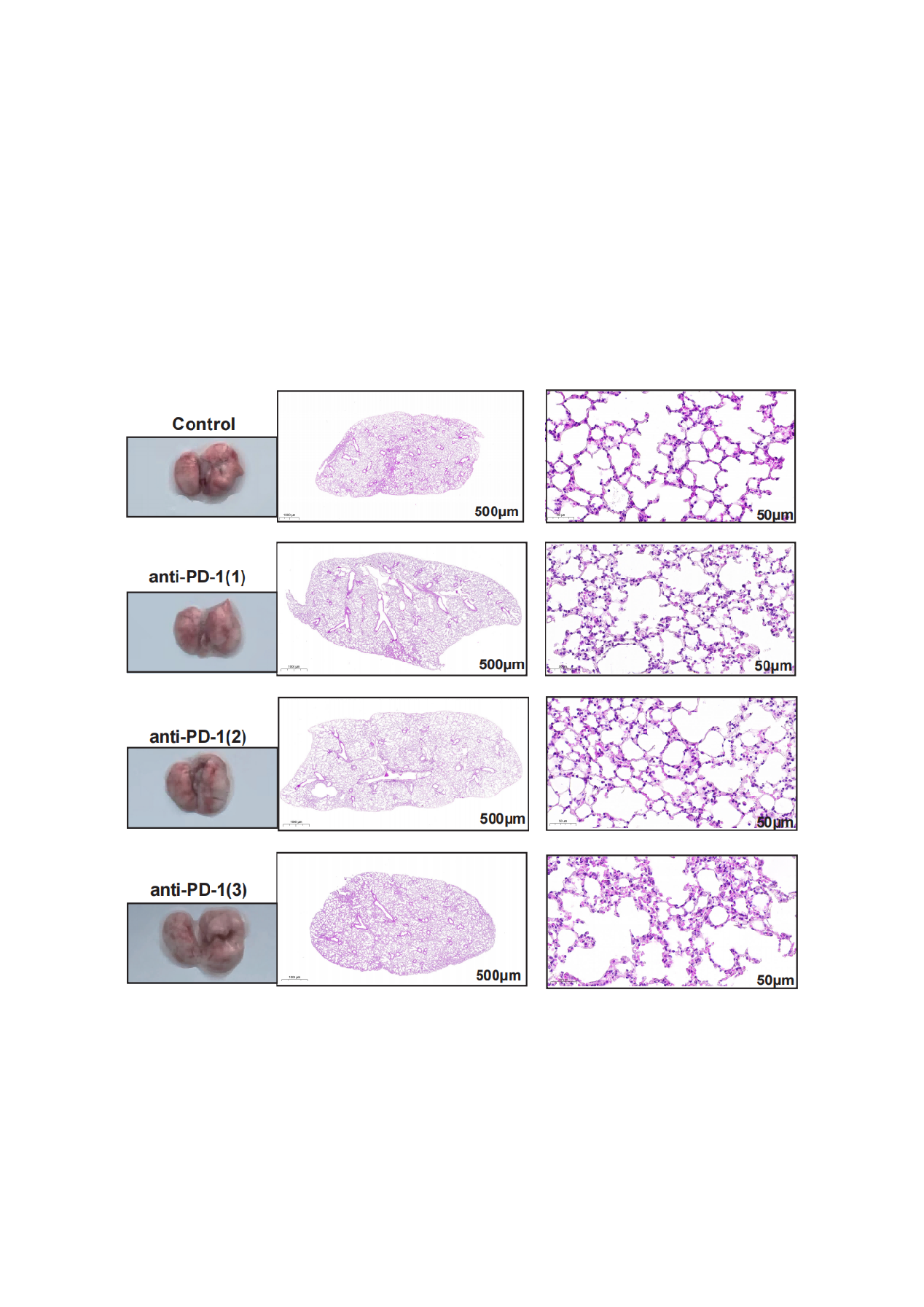


Supplementary Figure S4. Representative H&E (200 μm, 50 μm) images of the indicated mouse lung sections from mice treated with aPD-1 alone.


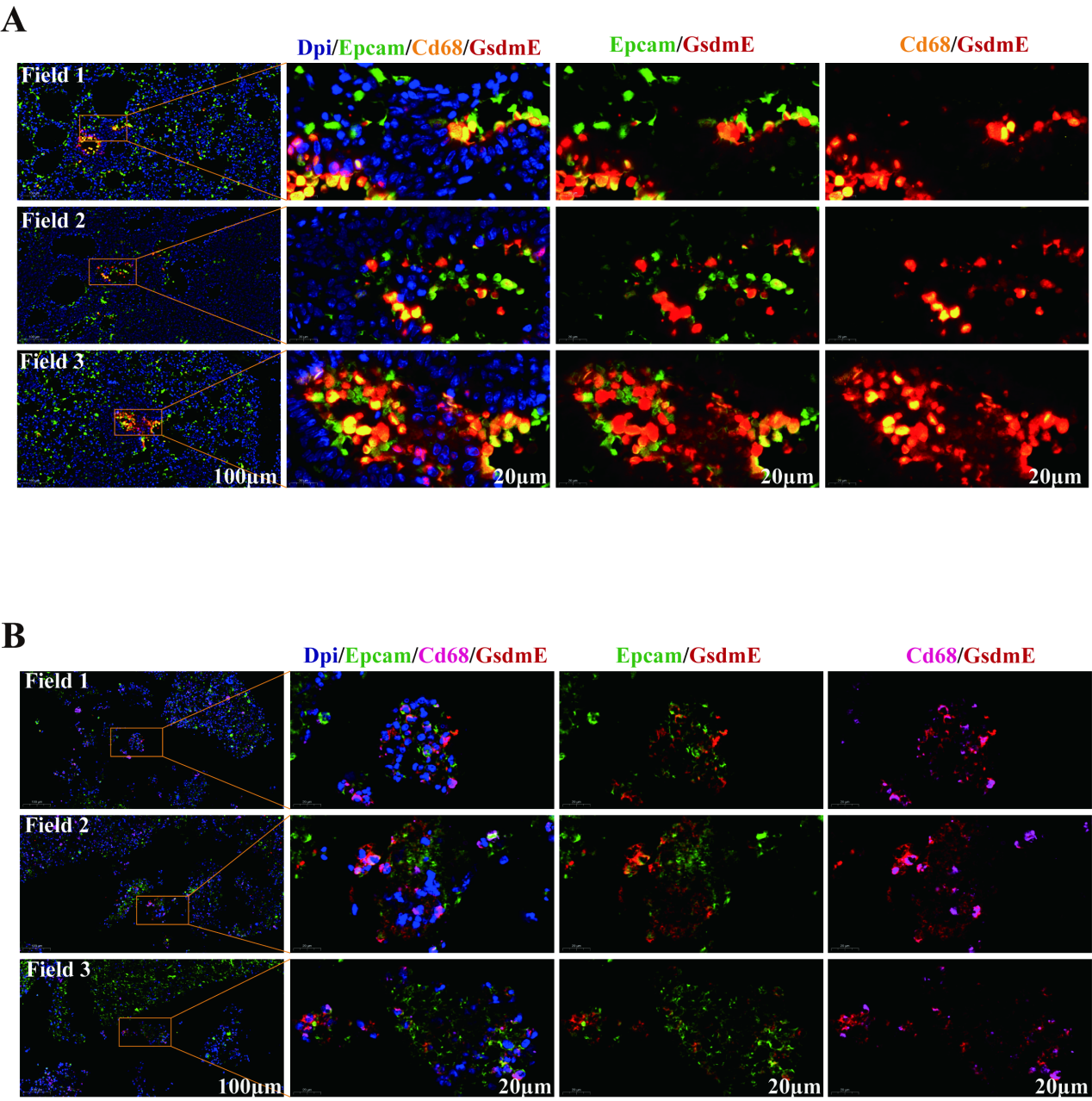


Supplementary Figure S5. Immunofluorescence staining of lung tissue from mice (A) and ICI-LI patients (B) with an anti-cleaved GSDME antibody.
