# Supplementary figures and images for: Anti-PD-1 exacerbates bleomycin-induced lung injury in mice via Caspase-3/GSDME-mediated pyroptosis
Source: Cell Death Dis. 2025 Jan 6;16(1):3. doi: 10.1038/s41419-024-07319-9 (PMC11704276; doi:10.1038/s41419-024-07319-9)

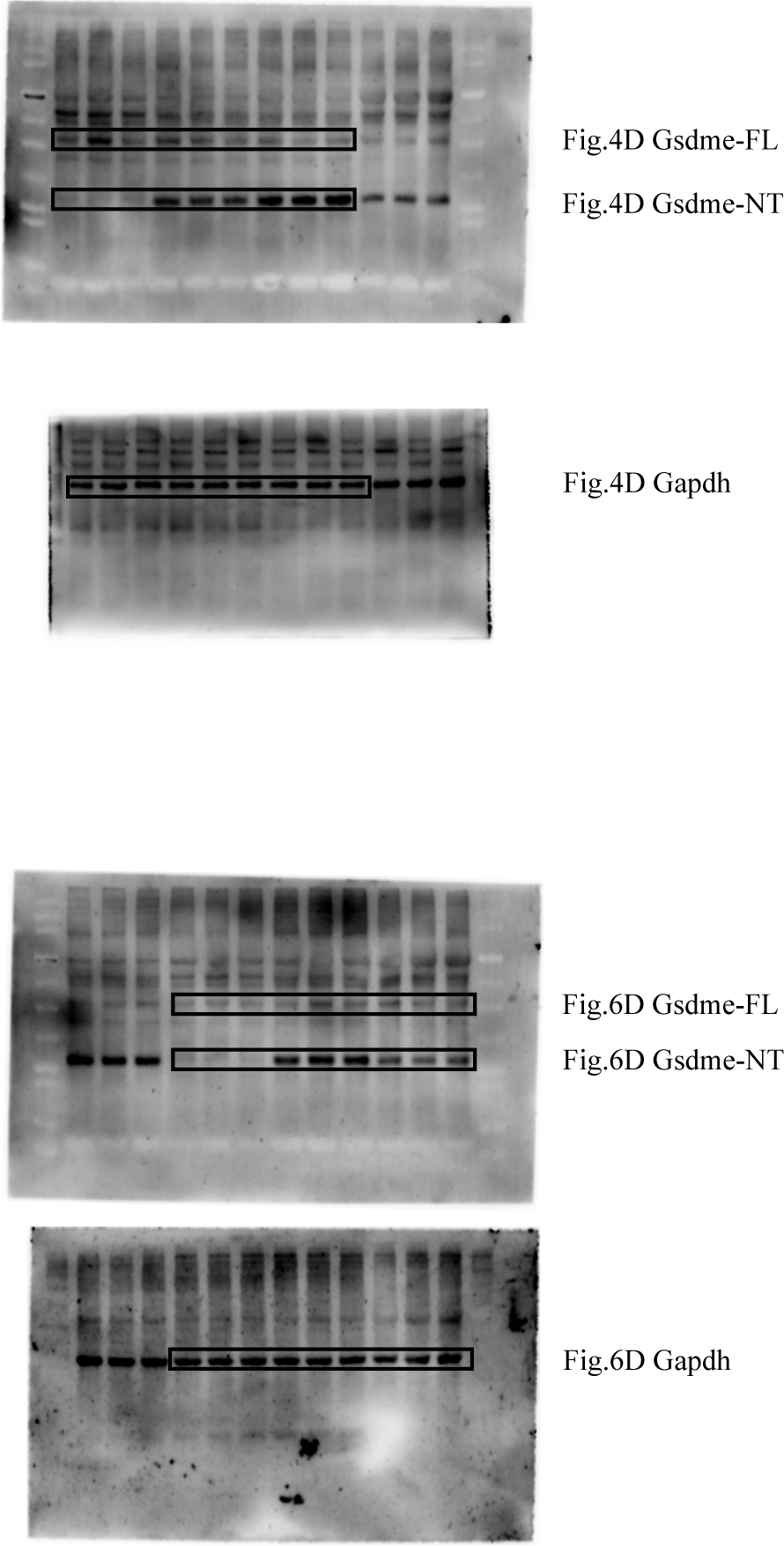

Supplement: Supplementary file 2 — Full and uncropped western blots [file 41419_2024_7319_MOESM2_ESM.tif]
